# Healthy School, Happy School: Design and Protocol for a Randomized Clinical Trial Designed to Prevent Weight Gain in Children

**DOI:** 10.5935/abc.20170072

**Published:** 2017-06

**Authors:** Daniela Schneid Schuh, Maíra Ribas Goulart, Sandra Mari Barbiero, Caroline D’Azevedo Sica, Raphael Borges, David William Moraes, Lucia Campos Pellanda

**Affiliations:** 1 Instituto de Cardiologia / Fundação Universitária de Cardiologia (IC/FUC); Porto Alegre, RS - Brazil; 2 Universidade Federal de Ciências da Saúde de Porto Alegre (UFCSPA); Porto Alegre, RS - Brazil

**Keywords:** Schools, Health Promotion, Health Behavior, Obesity, Motor Activity, Diet, Food and Nutrition, Body Weight, Prevention & Control

## Abstract

**Background:**

Schools have become a key figure for the promotion of health and obesity interventions, bringing the development of critical awareness to the construction and promotion of a healthy diet, physical activity, and the monitoring of the nutritional status in childhood and adolescence.

**Objectives:**

To describe a study protocol to evaluate the effectiveness of an intervention designed to improve knowledge of food choices in the school environment.

**Methods:**

This is a cluster-randomized, parallel, two-arm study conducted in public elementary and middle schools in Brazil. Participants will be children and adolescents between the ages of 5 and 15 years, from both genders. The interventions will be focusing on changes in lifestyle, physical activities and nutritional education. Intervention activities will occur monthly in the school’s multimedia room or sports court. The control group arm will receive usual recommendations by the school. The primary outcome variable will be anthropometric measures, such as body mass index percentiles and levels of physical activity by the International Physical Activity Questionnaire.

**Results:**

We expect that after the study children will increase the ingestion of fresh food, reduce excessive consumption of sugary and processed foods, and reduce the hours of sedentary activities.

**Conclusion:**

The purpose of starting the dietary intervention at this stage of life is to develop a knowledge that will enable for healthy choices, providing opportunities for a better future for this population.

## Introduction

The increased prevalence of obesity and its complications reinforces the global need for improved prevention strategies.^1-3^ In Brazil, population-based surveys indicate that overweight was present in 6% of children between 5 and 9 years in 1974-1975, rising steeply to 34.8% in 2008-2009.^4^ Globally, overweight in children increased 47.1% over the past 20 years.^5^ In 2010, it was estimated that overweight and obesity were responsible for 3.4 million deaths worldwide.^6^ Chronic diseases remain a public health challenge in Brazil. The medical costs associated with diseases related to overweight and obesity are substantial in Brazil, reaching nearly US$ 2.1 billion annually.^7^

Overweight in children and adolescents generates great concern because it is a risk factor for the development of hypertension, type 2 diabetes mellitus, dyslipidemia and other cardiovascular risk factors,^8,9^ which, if not prevented or treated at an early age, tend to persist during adulthood.^10^

Nutritional intervention studies have shown a positive effect on preferences for healthy foods and a decrease in daily consumption of sugary drinks.^11,12^ Permanent changes in diet quality, energy intake and physical activity demand preventive actions.^13^ On that account, promotion of a healthy diet, physical activity, and monitoring of the nutritional status in childhood and adolescence are essential elements in public health. Being an educational environment that contributes to build personal values, schools become a key figure for health promotion and obesity interventions, bringing the development of critical awareness to the construction and modification of eating habits.^14,15^

A number of international agencies, such as the Centers for Disease Control and Prevention (CDC) and the Institute of Medicine (IOM), launched campaigns with guidelines for health promotion in schools aiming to address the epidemic of obesity and its consequences.^16-18^ In Brazil, the School Health Program is designed to promote the comprehensive health care of public school students and is structured in four blocks that seek to: assess health conditions; perform actions of prevention and promotion of health conditions; promote continuing education for professionals and the young; present evaluation and monitoring of health conditions of the students. The responsibility for planning and carrying out these actions is upon the primary care health team, and the objective is to integrate the educational system and the Brazilian Unified Health System (SUS).^19^ However, this government action does not have coverage of all schools in the country yet.

In order to implement educational interventions in a large scale, it is important to adequately test their effectiveness. It is also important to look for simple and low-cost alternatives that can reach the largest possible number of schools. Improving the knowledge about food choices may be an important basis for children to acquire and maintain a healthy lifestyle from an early age, and possibly to sustain these healthy habits in subsequent stages of life.

Thus, the purpose of this study protocol is to evaluate the effectiveness of an intervention designed to improve knowledge of food choices in the school environment.

## Methods

This protocol is reported according to the SPIRIT (Standard Protocol Items: Recommendations for Interventional Trials) statement.

### Study design

#### Overview

**Figure 1:**
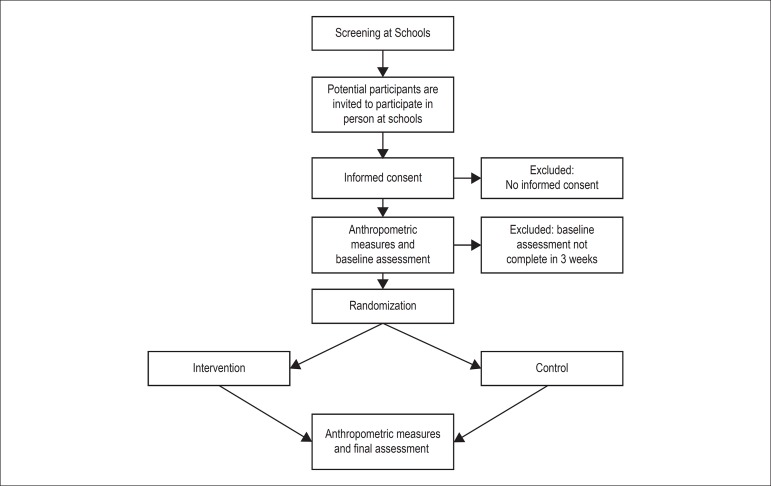
Flowchart of the study phases (enrolment, intervention allocation, and fnal assessments).

This is a cluster-randomized parallel two-arm study conducted in Brazil. The units of observation are individual children, and the units of randomization are schools. Randomization will be performed at the school level to avoid contamination. After the baseline assessment, enrolled schools are randomized to one of two study arms: the intervention arm focusing on changes in lifestyle, and the control group arm, that receives usual recommendations by the health care team. A summary of the study design, interventions and timelines is shown in Figure 1.

The primary outcome for the participants is change in body mass index (BMI = kg/m^2^), and the secondary outcomes are behaviors related to healthy eating, increased preferences for fruits and vegetables, increased physical activity and reduced screen time. The Institutional Research Ethics Committee approved the protocol for the study, which is registered in the Brazilian Registry of Clinical Trials, Register Number RBR-97bztb, and named “Intervention program for health promotion in schools of public elementary school in the state of Rio Grande do Sul: randomized clinical study”. The Universal Trial Number of this study is U1111-1155-7731.

#### Inclusion criteria

Children between the ages of 5 and 15 years, from both genders, enrolled in the public schools participating in this study, attending from the 1st to the 9th grades of the elementary and middle school will be eligible for the study (Table 1). The child and parent(s) or legal guardian(s) are required to sign the assent term and the informed consent.

**Table 1:** Inclusion criteria and assessment methods.

Inclusion criteria	Assessment method
Age 5-15 years	In person screening
Enrolled in one of the participant schools, attending a course from the 1^st^ to the 9^th^ grade of the elementary and middle school.	Electronic school review
Agreeing to participate in all the meetings of the study	In person screening

#### Exclusion criteria

Children are excluded if they have conditions or other circumstances that could interfere with participation in the measurements or the interventions or if the parent does not give or is unable to give consent or the child does not assent. Participants are also excluded if they do not complete baseline assessments in 3 weeks.

#### Screening and recruitment

The screening and recruitment activities will be developed during the course of 4 weeks. During the first week, the school electronic files of student enrollment will be consulted to identify potential participants (the eligibility criteria can be seen in Table 1). During the following two weeks, recruitment letters will be sent to the student's guardians, with explanation of the study and attached Informed Consent Form. During the fourth week, individuals who agree to participate in the study will undergo anthropometric assessment, provided that there is no impediment for physical evaluation.

#### Randomization

Cluster randomization will be performed with distribution of two schools for the control group and two schools for the intervention group. A biostatistician who does not have direct contact with study participants will generate the random allocation sequences using a computerized random number generator. After the inclusion of each cluster, the allocation of that particular cluster will be provided to the study coordinator. Due to the characteristics of the intervention, it is not possible to mask participants or interventionists to group assignment. There will be no crossover between study arms, but the intervention will be offered to the control group at the end of the study, if proved to be effective.

#### Assessments

Measures are conducted at baseline (month one) and post-treatment (month 9).

#### Anthropometric measures

Weighing electronic scales with a maximum capacity of 150 kg and precision of 100 g, properly checked for tare weight, will be used for weight measures. The individual will be weighted barefoot and wearing light clothes. A metallic measuring tape with a capacity of 2 m/0.1 cm, set in an existing flat wall in the room, will be used to measure height, with the individual in the upright position, during maximum inspiration, barefoot and with empty pockets. These data will be used for the following calculation of BMI, obtained by weight, measured in kilograms, divided by the square of the height, measured in meters (kg/m^2^). That will be calculated and nutritional status will be classified, both using the *Anthro Plus* software, according to the reference of the World Health Organization (WHO) 2006/2007.

#### Dietary intake

The collection of dietary data, referring to the eating habits of the participants, will be assessed by a Food Frequency Questionnaire (FFQ),^20^ previously validated for the study population, designed to collect information on the frequency of food consumption and/or food groups for further association with other study variables, such as lifestyle and anthropometric measurements.

#### Physical activity

International Physical Activity Questionnaire (IPAQ)^21,22^ will be used for all ages to classify the level of physical activity, in spite of the fact this instrument has being validated only for adults and adolescents, since there is not yet a validated questionnaire that can suit the needs of this study and adequately classify physical activity levels in Brazilian children.

#### Screen Time

Screen time refers to the amount of time individuals spend in front of television, video games and computer, and is considered to be a sedentary activity. It will be measured by hours per day and number of days in the week of screen time.

#### Knowledge of healthy habits

The acquisition of knowledge about healthy habits will be evaluated through a questionnaire validated for age,^23^ consisted of knowledge about food and healthy activities in daily life.

#### Demographics measures

During baseline assessment, parents will be asked about a series of demographic data included in the Brazilian Economic Classification Criterion by the Brazilian Association of Research Companies,^24^ which includes: age; educational attainment of the family head; questions about household appliances and other family properties; street paving and treated water in the house.

#### Sample size

The sample size was calculated to detect a difference in 0.2 kg/m^2^ on BMI, with a standard deviation of 0.05, statistical power of 90% and alpha error of 0.05. An estimate of mean 19 kg/m^2^ is used as obtained from our pilot study. A number of 99 participants per group was estimated using these parameters. To compensate for losses, the sample size will be increased by 10%.

#### Planned data analyses

Collected data will be entered and analyzed using the Statistical Package for Social Sciences, version 17.0. Quantitative variables will be expressed as mean and standard deviation in the presence of normal distribution or median and interquartile range in the presence of asymmetric distribution. Qualitative variables are expressed as absolute and relative frequencies.

Adjusted analysis for primary and secondary outcomes will be performed using generalized estimating equations (GEE). The comparison between secondary outcomes will be performed using the nonparametric Wilcoxon test.

The level of significance for all tests will be 95% (α = 0.05) and will follow the intention to treat principle. P-values will be reported up to three decimal places with p-values < 0.005 reported as p < 0.005. The outcomes will be evaluated by a blind adjudicator.

#### Interventionists

The interventions in this study will be delivered by the nutritionists of the Children and Adolescents Cardiovascular Prevention Group (PREVINA),^25^ nutrition graduate students and health professional contributors employed by the City Hall (psychologists, physical education teachers, nurses). All interventionists will undergo extensive training on: intervention protocol; overall intervention objectives, content and format; and specific instructions for each intervention session.

#### Intervention description

Intervention activities will occur monthly in the school multimedia room or sports court (Table 2). All activities will be offered in different school shifts and schedules so that all students in all classes can participate.

**Table 2:** Description of intervention activities

Intervention	Type	Description	Moment
Presentation of the program to students, parents and teachers.	Seminar	A seminar approaching the following topics will be conducted: epidemiological data on obesity and noncommunicable disease risk factors; dyslipidemia tracking; importance of school intervention; presentation of the activities that will be developed during the school year; reading and clarification of the Informed Consent Form.	1st Month
Knowing what we eat.	Seminar	The seminar will succinctly address the composition of food, approaching macronutrients, fibers and food groups. As a task for the week, each class should study the vitamins and prepare a poster on that topic during class time. As a homework assignment, all students should create, with the help of parents, a list of five fruits and six vegetables they like to eat. Three of them should be cooked vegetables and three of them raw vegetables.	2nd Month
The importance of water.	Seminar	The seminar will address the importance of water in our health and the consequences of high consumption of sugary drinks. Soft drink consumption has increased in recent decades in Latin America, and that is being referred as a contributor to the population weight gain. Many schools sell soft drinks in the cafeteria and some children end up consuming them daily without parents' knowledge.The “Week without soda” challenge will be launched, in which children, parents and teachers will commit to spend a week without drinking soft drinks, which will require the family interaction and commitment for the accomplishment of the goal.	3rd Month
Revolution in the kitchen: first you taste, and then you like it.	Workshop	The workshop aims to propose an interaction among students, teachers, kitchen staff and the food. Various whole foods (fruits, vegetables, breads, cakes, cookies) will be placed on a table. Participants will be blindfolded and will have to randomly taste a food, describe their sensory characteristics and perceptions (whether it is soft or hard, if it has good or bad smell, if they like it or not) and try to guess what it is. After that, participants will be invited to prepare their own snack with some of the vegetables they planted earlier in the year, according to their preferences. As homework assignment, students will be asked to help parents to make the salad for the family for a day, starting with the choice of food in the supermarket. During class time, teachers, helped by a nutritionist, will work on the dynamics of the traffic light food, where the food is classified according to the colors of the traffic light. Green represents foods we should eat daily; yellow foods that can be eaten more than once a week, but in moderation; and red are the forbidden foods to eat daily, but which can be eaten on special occasions, like parties or the weekend. There will also be a seminar for parents in order to provide information and ideas for preparation of healthy snacks for school lunches best suited for the needs of children.	4th Month
Let's Move! Physical Activity in all moments!	Seminar	The seminar will address the importance of physical activity in our health, and it will present the physical activity programs that are available in the city. Activity ideas to do either outdoors or at home, alone or in groups, will be suggested. A challenge will be launched: “one disconnected day”, in which students, parents and teachers will be invited to reduce television and internet time to only two hours for a day. Reducing sedentary behaviors, such as spending too much time watching television and using the computer, appears to contribute to the reduction of daily calorie intake. Activity suggestions will be given for that day.	5th Month
Where does my food come from?	Workshop	The workshop will have the participation of a city vegetable producer who will teach students how to plant and take care of a small vegetable garden at school. In addition, each student will receive seeds of green seasoning (parsley) to plant in a little vase previously decorated during arts education class.	6th Month
Milk every day!	Seminar	The seminar will address the importance of milk and dairy products in our diet and the recommended amounts of ingestion. It will also address certain disorders related to milk digestion, such as lactose intolerance and allergy to cow's milk protein.	7th Month
Bullying: we have to talk about it!	Seminar	A psychologist will conduct the seminar and address the meaning of the theme, bullying types, and what to do if you fall victim. There will be a special meeting with teachers to answer questions.	8th Month
German dances course	Workshop	It will be offered fortnightly lessons of German dances in school during one school year. This course also aims to rescue the traditions and culture of the city, which was settled mostly by Germans.	Ongoing activity during the year

#### Usual care comparator

Participants randomized to the control group will not receive any guidance during the study. Children will receive the usual care and recommendations through the school and health authorities.

Should the intervention prove to be effective, at the end of the study, the institutions allocated to the control group will receive all intervention activities if they wish.

## Discussion

This randomized clinical trial is intended to help filling a gap in the literature regarding simple, low-cost and effective interventions to deal with the epidemic of obesity and overweight in developing countries. Numerous studies show that overweight and obesity rates in the young in Latin America bring important economic and health consequences.^26^ In spite of the need for individual approaches for children who are already overweight or obese, the international consensus is that prevention is the most realistic approach and the best value for money.^27^ It is therefore necessary to develop preventive interventions that can reach a larger number of children.

Facilitators and barriers for the development of healthy habits should be considered when designing a childhood overweight program. Adaptation to local culture and reality should also be a concern. One of the most important challenges of this study will be changing the approach to nutrition and physical activity. On that account, we designed interventions that include parents, teachers and students aimed at creating a positive impact on the health of children and adolescents.

The young need appropriate information to make healthy choices and change their sedentary behavior, but their parents and teachers are not always prepared to give that information. Therefore, involvement of trained health professionals specialized in the field is necessary in order to adequately provide that information. Given that children are exposed to the environment we create for them and that in Brazil they usually spend about 25 hours a week in school, it is important to design actions that seek to improve the school environment and create a healthy growth strategy.

The study also has some limitations, that must be addressed in future work. First, there is a possibility of cross-contamination of participants in the two intervention arms, since both interventions are delivered in a small city. Second, the trial does not evaluate the pubertal maturation, whose changes may impact on the body composition in childhood and early adolescence, such as weight gain in girls and a decline of body fat in pubertal boys.

In conclusion, we have described the basic rationale and design of the ongoing Healthy School, Happy School cluster-randomized trial. The study intervention aims to increase the ingestion of fresh food, reduce excessive consumption of sugary and processed foods, and reduce the hours of sedentary activities. The purpose of starting the dietary intervention at this stage of life is to develop a knowledge that will enable for healthy choices, providing opportunities for a better future for this population.
